# Functional Hemispheric (A)symmetries in the Aged Brain—Relevance for Working Memory

**DOI:** 10.3389/fnagi.2018.00058

**Published:** 2018-03-12

**Authors:** Madalena Esteves, Ricardo Magalhães, Paulo Marques, Teresa C. Castanho, Carlos Portugal-Nunes, José M. Soares, Armando Almeida, Nadine C. Santos, Nuno Sousa, Hugo Leite-Almeida

**Affiliations:** ^1^Life and Health Sciences Research Institute (ICVS), School of Medicine, University of Minho, Braga, Portugal; ^2^ICVS/3B’s—PT Government Associate Laboratory, Braga, Portugal; ^3^Clinical Academic Center — Braga, Braga, Portugal

**Keywords:** laterality, N-back, aging, fMRI, superior parietal

## Abstract

Functional hemispheric asymmetries have been described in different cognitive processes, such as decision-making and motivation. Variations in the pattern of left/right activity have been associated with normal brain functioning, and with neuropsychiatric diseases. Such asymmetries in brain activity evolve throughout life and are thought to decrease with aging, but clear associations with cognitive function have never been established. Herein, we assessed functional laterality during a working memory task (N-Back) in a healthy aging cohort (over 50 years old) and associated these asymmetries with performance in the test. Activity of lobule VI of the cerebellar hemisphere and angular gyrus was found to be lateralized to the right hemisphere, while the precentral gyrus presented left > right activation during this task. Interestingly, 1-Back accuracy was positively correlated with left > right superior parietal lobule activation, which was mostly due to the influence of the left hemisphere. In conclusion, although regions were mostly symmetrically activated during the N-Back task, performance in working memory in aged individuals seems to benefit from lateralized involvement of the superior parietal lobule.

## Introduction

The human brain presents several marked structural asymmetries such as the Yakovlevian torque (Toga and Thompson, 2003; Hugdahl, 2011) or the asymmetry of the planum temporale (Takao et al., 2011), as well as chemical left/right unbalances in dopaminergic (Glick et al., 1982), noradrenergic (Oke et al., 1978) and opioidergic (Watanabe et al., 2015) systems.

It is known since the early observations by Mark Dax and Paul Broca (Broca, 1861; see also Roe and Finger, 1996; Manning and Thomas-Antérion, 2011) that the brain also distributes its functional load in an asymmetrical fashion. Numerous imaging studies have confirmed these observations reporting an asymmetric recruitment of brain regions not only for language processing but also for emotion (Costanzo et al., 2015), motivation (Poole and Gable, 2014; Hughes et al., 2015), memory (Otsuka et al., 2008; Brambilla et al., 2015) and even general intelligence (Santarnecchi et al., 2015). A relevant body of literature also describes abnormal lateralization associated with neuropsychiatric diseases such as autism (Conti et al., 2016), schizophrenia (Royer et al., 2015) and dyslexia (Leonard and Eckert, 2008; Altarelli et al., 2014) suggesting that fine-tuned asymmetry is fundamental for, or at least reflects, proper functioning. In aged individuals, marked structural asymmetries have been identified (Esteves et al., 2017) and functional studies have systematically reported decreases in lateralization; such has been observed in word encoding/retrieval (Cabeza et al., 1997; Madden et al., 1999; Stebbins et al., 2002), working memory (Reuter-Lorenz et al., 2000), face recognition (Grady et al., 2002), inhibitory control (Nielson et al., 2002), risk-taking (Lee et al., 2008) and error processing (Zhu et al., 2010). Such bilateral activity pattern has been hypothesized to result from a compensatory recruitment (Cabeza, 2002) and, in fact, as cognitively efficient older performers recruit additional contra-lateral networks (Cabeza et al., 2002; Erickson et al., 2007) symmetrical activation could be a correlate of good cognitive aging.

Nonetheless, the differential left/right hemisphere involvement in working memory, both in the general population and in distinct age groups, is still not clear. We hypothesize that left and right areas are involved in working memory in different degrees in an older population. Herein, we evaluated functional laterality in an aged cohort using a cognitively stringent working memory task—N-Back (Vermeij et al., 2012)—in two different memory loads (1- and 2-back tasks), aiming to assess the validity of our hypothesis.

## Materials and Methods

### Ethics Statement

This study was performed in accordance with the Declaration of Helsinki (59th amendment) and approved by national and local ethics review boards (Hospital de Braga, Centro Hospitalar do Alto Ave and Unidade Local de Saúde do Alto Minho) and by the national data protection entity (Comissão Nacional de Protecção de Dados). All volunteers signed informed consent and all medical and research professionals who had access to participants’ identity signed a Statement of Responsibility and Confidentiality.

### Subjects

The sample used in the present study was recruited from the Switchbox project. Briefly, a large sample, representative of the older Portuguese population in terms of sex and education, was cognitively characterized (*n* = 1051, after inclusion/exclusion criteria; subjects were randomly selected from the Guimarães and Vizela local area health authority registries (Costa et al., 2013; Santos et al., 2013, 2014)). For this study, we started with a subcohort of 60 participants; 31 participants were selected because they showed above-minimum accuracy in the N-back task (see below); these subjects displayed high overall cognitive performance (for further detail regarding the participants’ selection process, tests used for the cognitive assessment and the group formation, please consult (Marques et al., 2016)). All participants were right-handed.

### Task Design and Minimum Performance Definition

**Figure 1:**
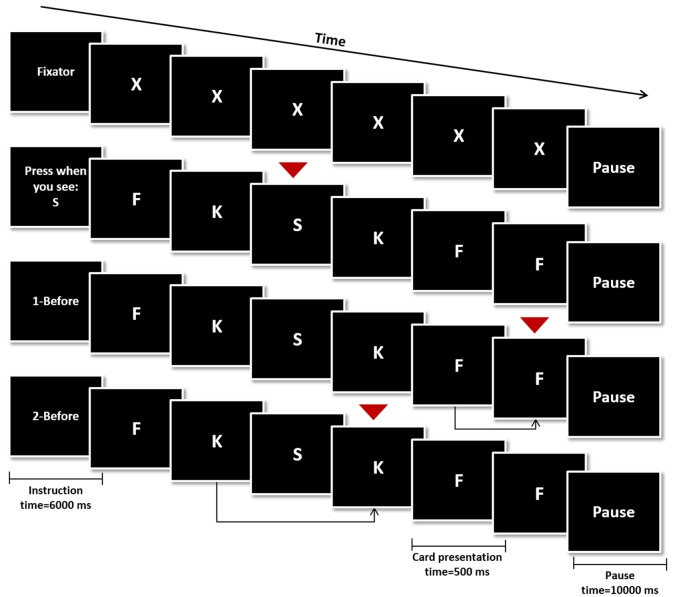
Schematic representation of the N-Back Task. The four conditions were initiated by an instruction card (6000 ms), followed by the presentation of 16 trial cards (500 ms) and ended with a pause card (10,000 ms). In the rest condition the letter “X” was presented in every trial and none was a target card (top). In the remaining conditions, target cards (red arrows) were a specific letter (0-Back—second row from the top), any card similar to the one shown in the previous trial (1-Back—third row from the top) or any card similar to the one shown two trials before (2-Back—bottom row).

During the fMRI session, participants performed a modified version of the verbal N-Back task. The task was composed by four different conditions: rest condition (Figure 1, top), 0-Back (Figure 1, second row from the top—control condition), 1-Back (Figure 1, third row from the top—low working memory load) and 2-Back (Figure 1, bottom—high working memory load). Four blocks of every condition (16 blocks in total) were pseudo-randomly distributed throughout the experiment. Each block was initiated by the presentation of an instruction card indicating the block condition (6000 ms) followed by 16 trial cards and ended with a pause card (10,000 ms). Each trial card contained a letter which was presented for 500 ms, followed by a fixation cross card for 2000 ms. Response was allowed up to the first 1900 ms of the fixation cross card, totaling 2400 ms of response time.

Participants were instructed to press a button if the trial card presented was a target card during the control and experimental conditions (0−, 1− and 2-Back). On 0-Back blocks, the target letter was presented in the instruction card and participants were instructed to press the button in trial cards showing the same letter. On 1-Back, the target was any letter equal to the one presented one trial before and on 2-Back, subjects should press the button if the letter corresponded to the one presented two trials before. Finally on the rest condition blocks participants were instructed to remain still, looking at the screen, without pressing any button while the letter “X” was presented in trial cards.

Inclusion of subjects in the fMRI analysis was dependent on N-Back performance. Accuracy (Acc) per block was calculated as Acc = 1−(inc/4), where inc = number of incorrect answers in each block (omissions and non-target button presses) and 4 is the maximum number of possible correct responses per block. The worst trial of each condition was excluded and subjects were included in the analyses if average Acc ≥ 0.5 (i.e., number of correct responses ≥ number of incorrect responses), in order to guarantee subject’s engagement in the task. Following these criteria 31 and 22 participants were included for1-Back and 2-Back analyses, respectively.

### Image Acquisition

Acquisitions were performed on a clinically approved Siemens Magnetom Avanto 1.5 T (Siemens Medical Solutions, Elangen, Germany) scanner in Hospital de Braga, using a 12-channel receive-only Siemens head coil. Two acquisitions were used in the current work: as a structural acquisition a T1-weighted magnetization-prepared rapid gradient echo (MPRAGE) sequence with repetition time (TR) = 2730 ms, echo time (TE) = 3.5 ms, flip angle (FA) = 7°, field of view (FoV) = 256 × 256 mm, with isotropic resolution of 1 mm and no slice-gap; the functional acquisition consisted of a T2-weighted Echo Planar Imaging (EPI) sequence, sensible to blood oxygen level dependent (BOLD) contrast, with TR = 2000 ms, TE = 30 ms, FA = 90°, FoV = 224 × 224 mm, in plane resolution = 3.5 × 3.5 mm, slice thickness = 4 mm, slice-gap = 0.48 mm slice gap, 30 interleaved ascending slices and with 456 volumes for a total of 15 min and 12 s acquisition time. The task was presented using the fully integrated fMRI system IFIS-SA (Invivo Corporation, Orlando, FL, USA) and the same system was used to record the subject key-press responses.

### fMRI and Statistical Analysis

Pre-processing of functional data was performed using SPM8^1^ and consisted of the following steps: slice timing correction, within subject registration of each volume to the first volume of the acquisition in order to correct for head movement, non-linear spatial normalization to MNI standard space, resampling to 2 mm isotropic resolution, spatial smoothing using an 8 mm FWHM Gaussian kernel and high-pass temporal filtering at 128 s.

The first-level analysis was performed using the General Linear Model (GLM) framework implemented in SPM. For the included participants, a total of 14 regressors were introduced in the model: one regressor modeling the instruction and pause cards; one regressor for the rest condition blocks; three regressors, one per experimental condition (0−, 1− and 2-Back) modeling the three included blocks; three regressors, one per experimental condition modeling the block with worst accuracy, excluding it from the analysis; six movement regressors. Contrasts of interest were set to create maps of increased or decreased activity in low working memory load (1- > 0-Back and 1- < 0-Back), high working memory load (2- > 0-Back and 2- < 0-Back) and high working memory load compared to low working memory load (2- > 1-Back and 2- < 1-Back). In the second-level analysis (group level) one-sample *t*-tests were performed for the above-mentioned contrasts and areas were considered active for each condition if the corresponding family wise error (FWE) corrected *p*-values were <0.05 at the voxel level.

Laterality assessment was performed based on the regions of the Automated Anatomical Labeling (AAL) atlas (Tzourio-Mazoyer et al., 2002) in which active voxels were found and were based on the magnitude signal change as previously described (Jansen et al., 2006). Briefly, mean maximum activation was calculated for each region of interest (ROI) as the mean of the 5% of voxels evidencing the highest T-score. Voxels with signal intensity change higher than 50% of the mean maximum activation were included.

Statistical analyses were performed on Matlab R2009b software (The MathWorks, Inc., Natick, MA, United States) and images were computed in Prism 6 (GraphPad Software Inc.) and MRIcro (Rorden and Brett, 2000). Independent and paired analyses were performed respectively for population comparisons and assessment of left/right differences in activation. Non-parametric tests were used whenever normality could not be confirmed. For linear regressions, laterality degree (LD) was calculated as *L* = *A*_L_ − *A*_R_, where *A*_L_ = left activation of the specified region on the specified contrast and *A*_R_ = right activation of the specified region on the specified contrast. *P*-value < 0.05 was considered the threshold for statistical significance and Bonferroni correction was applied whenever multiple comparisons were performed. Data is shown as mean ± standard deviation.

## Results

### Population Characterization

Thirty-one subjects showed above-minimum accuracy for inclusion in 1- vs. 0-Back analysis respectively and 22 out of these 31 were also included for 2- vs. 0-Back and 2- vs. 1-Back analyses. Included individuals significantly differed from the remaining population of 29 subjects in terms of gender (32.258 vs. 57.143% female), age (60.290 ± 7.708 vs. 69.035 ± 7.356 years old; *Z* = 3.861; *p* < 0.001; Cohen’s *d* = 1.161) and education (8.484 ± 4.545 vs. 5.000 ± 3.162 years of formal education; *Z* = 3.515; *p* < 0.001; Cohen’s *d* = 0.890).

### N-Back-Associated BOLD Response and Patterns of Asymmetry

**Figure 2:**
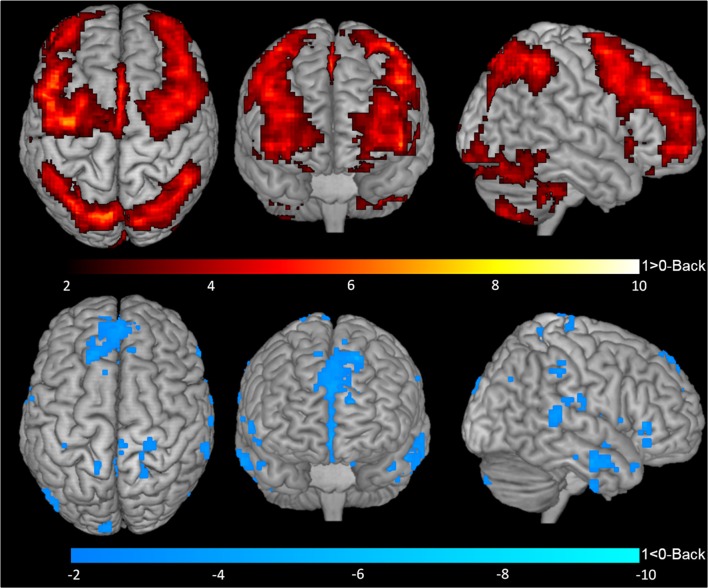
Blood oxygen level dependent (BOLD) response during 1-Back performance. Representation of 1-Back performance functional activation (1-Back > 0-Back—top) and deactivation (1-Back < 0-Back—bottom) in axial (left), coronal (middle) and sagittal (right) views. Hotter and colder colors represent respectively increased activation and deactivation.

**Figure 3:**
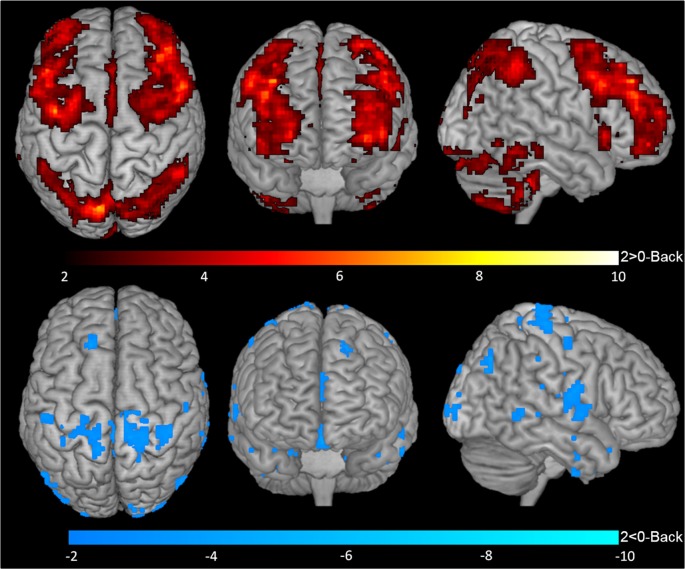
BOLD response during 2-Back performance. Representation of 2-Back performance functional activation (2-Back > 0-Back—top) and deactivation (2-Back < 0-Back—bottom) in axial (left), coronal (middle) and sagittal (right) views. Hotter and colder colors represent respectively increased activation and deactivation.

One-sample *t*-tests for contrasts 1-Back > 0-Back (Figure 2, top), 1-Back < 0-Back (Figure 2, bottom), 2-Back > 0-Back (Figure 3, top) and 2-Back < 0-Back (Figure 3, bottom) showed that BOLD response during N-Back performance followed the expected patterns (Owen et al., 2005). No activated/deactivated areas were found in the 2- vs. 1-Back contrasts.

A total of 20 ROIs were found to be either activated (1-Back > 0-Back) or deactivated (1-Back < 0-Back) in the 1-Back condition (Table 1, Figure 2). These comprised the superior and inferior parietal lobules, Crus I of cerebellar hemisphere, orbital part of the middle frontal gyrus, lobules VI and VII of cerebellar hemisphere, middle frontal and precentral gyri, Crus II of cerebellar hemisphere, supplementary motor area, pars triangularis of the inferior frontal gyrus, lobule VIII of cerebellar hemisphere, superior frontal and angular gyri, precuneus, insula, rolandic operculum, medial frontal and superior temporal gyri and medial orbitofrontal cortex.

**Table 1:** Activations found in the N-Back task.

Cluster		Peak		Coordinates			Hemisphere	AAL Area
α	Size	α	*Z*	*X*	*Y*	*Z*
**1->0-Back**
<0.001	191	<0.001	7.033	−30	−66	51	Left	Superior parietal lobule
		0.001	5.464	−15	−72	57	Left	Superior parietal lobule
		0.002	5.433	−6	−69	57	Left	Precuneus
<0.001	59	<0.001	6.416	−30	−3	60	Left	Precentral gyrus
		<0.001	5.693	−39	−3	63	Left	precentral gyrus
		0.001	5.592	−24	9	57	Left	Middle frontal gyrus
<0.001	174	<0.001	6.354	−48	9	33	Left	Precentral gyrus
		<0.001	6.021	−45	24	39	Left	Middle frontal gyrus
		<0.001	5.940	−42	3	42	Left	Precentral gyrus
<0.001	78	<0.001	5.893	3	15	51	Right	Supplementary motor area
		0.004	5.273	−3	0	60	Left	Supplementary motor area
<0.001	46	<0.001	5.809	6	−78	−15	N/A	Lobule VI of vermis
		0.006	5.180	6	−81	−30	Right	Crus II of cerebellar hemisphere
<0.001	60	<0.001	5.671	−42	−45	45	Left	Inferior parietal lobule
		0.001	5.546	−51	−42	48	Left	Inferior parietal lobule
<0.001	123	<0.001	5.664	39	−60	51	Right	Angular gyrus
		0.001	5.521	33	−57	57	Right	Superior parietal lobule
		0.002	5.395	51	−45	48	Right	Inferior parietal lobule
<0.001	14	<0.001	5.640	−6	−75	−24	Left	Crus I of cerebellar hemisphere
<0.001	11	0.001	5.619	−48	48	−3	Left	Middle frontal gyrus. orbital part
		0.045	4.793	−45	48	6	Left	Inferior frontal gyrus. pars triangularis
<0.001	24	0.001	5.596	30	3	60	Right	Superior frontal gyrus
<0.001	26	0.001	5.592	39	15	51	Right	Middle frontal gyrus
		0.003	5.318	51	9	36	Right	Precentral gyrus
0.001	6	0.001	5.440	36	24	0	Right	Insula
<0.001	24	0.002	5.368	42	30	36	Right	Middle frontal gyrus
		0.012	5.052	45	39	30	Right	Middle frontal gyrus
<0.001	26	0.002	5.360	27	−72	−21	Right	Lobule VI of cerebellar hemisphere
<0.001	20	0.004	5.264	12	−75	51	Right	Superior parietal lobule
<0.001	29	0.004	5.263	−36	−72	−21	Left	crus I of Cerebellar hemisphere
		0.012	5.053	−30	−69	−27	Left	Crus I of cerebellar hemisphere
0.007	2	0.011	5.070	−33	−36	−42	Left	Lobule VIII of cerebellar hemisphere
0.002	4	0.014	5.016	42	3	63	N/A	Undefined
0.015	1	0.028	4.890	33	63	15	N/A	Undefined
0.015	1	0.033	4.855	36	60	−9	Right	Middle frontal gyrus. orbital part
0.015	1	0.041	4.810	42	3	54	Right	Middle frontal gyrus
0.015	1	0.042	4.808	−30	−66	−42	Left	lobule VIIB of Cerebellar hemisphere
0.015	1	0.042	4.806	45	51	−6	Right	Middle frontal gyrus. orbital part
0.015	1	0.043	4.803	−36	54	18	Left	Middle frontal gyrus
**1-<0-Back**
<0.001	49	<0.001	6.474	39	−18	9	Right	Insula
		<0.001	6.426	42	−12	15	Right	Rolandic operculum
<0.001	7	0.004	5.281	9	−54	18	Right	Precuneus
		0.004	5.248	9	−51	27	Right	Precuneus
<0.001	19	0.006	5.172	−6	−54	24	Left	Precuneus
		0.009	5.100	−12	−57	18	Left	Precuneus
0.015	1	0.017	4.987	42	−12	−3	Right	Insula
0.003	3	0.019	4.963	−9	54	12	Left	Medial frontal gyrus
0.015	1	0.027	4.895x	−42	−33	15	Left	Superior temporal gyrus
0.007	2	0.039	4.819	−9	48	3	Left	Medial frontal gyrus
0.015	1	0.046	4.788	−3	54	−9	Left	Medial orbitofrontal cortex
0.015	1	0.047	4.784	0	45	−9	Left	Medial orbitofrontal cortex
**2->0-Back**
<0.001	9	<0.001	5.902	−48	9	33	Left	Precentral gyrus
<0.001	14	<0.001	5.693	−9	−69	60	Left	Precuneus
0.001	4	0.001	5.561	39	42	39	Right	Middle frontal gyrus
<0.001	6	0.001	5.438	42	45	27	Right	Middle frontal gyrus
<0.001	8	0.002	5.343	33	24	0	Right	Insula
<0.001	7	0.003	5.337	−30	24	0	Left	Insula
<0.001	8	0.004	5.245	51	9	42	Right	Precentral gyrus
0.001	4	0.008	5.129	−42	3	57	Left	Precentral gyrus
0.014	1	0.009	5.099	−30	−27	−33	Left	Lobule IV. V of cerebellar hemisphere
0.001	4	0.010	5.092	54	21	36	Right	Inferior frontal gyrus. pars opercularis
<0.001	7	0.011	5.064	−9	−75	−30	Left	Crus I of cerebellar hemisphere
<0.001	10	0.011	5.063	−48	30	30	Left	Middle frontal gyrus
0.014	1	0.015	5.008	−30	9	66	N/A	Undefined
0.014	1	0.018	4.977	−33	57	−3	Left	Middle frontal gyrus. orbital part
0.014	1	0.022	4.937	−24	−69	45	Left	Superior parietal lobule
0.005	2	0.023	4.927	−33	−33	−36	Left	Lobule VI of cerebellar hemisphere
<0.001	6	0.025	4.910	−27	−66	51	Left	Superior parietal lobule
0.014	1	0.026	4.900	36	27	−6	Right	Inferior frontal gyrus. pars orbitalis
0.005	2	0.030	4.869	−30	−57	51	Left	Inferior parietal lobule
0.014	1	0.036	4.839	−48	24	45	N/A	Undefined
0.014	1	0.036	4.838	−24	−72	36	Left	Superior occipital
0.014	1	0.036	4.836	42	21	3	Right	Inferior frontal gyrus. pars triangularis
0.014	1	0.039	4.821	−42	0	42	Left	Precentral gyrus
0.014	1	0.044	4.795	45	45	18	Right	Middle frontal gyrus
0.014	1	0.046	4.785	−45	51	−3	Left	Middle frontal gyrus. orbital part
**2-<0-Back**
<0.001	5	0.018	4.976	−9	−54	24	Left	Precuneus

*Statistically significant BOLD changes found in the 1- and 2-Back (vs. 0-Back) contrasts. α, FWE corrected p-values; Z, z-score values of t-statistics; AAL, Automated Anatomical Labeling atlas*.

During the 2-Back part of the task, 14 regions were involved (Table 1, Figure 3), namely superior and inferior parietal lobules, Crus I of cerebellar hemisphere, orbital part of the middle frontal gyrus, lobule VI of cerebellar hemisphere, precentral gyrus, pars opercularis of the inferior frontal gyrus, middle frontal gyrus, pars triangularis of the inferior frontal gyrus, superior occipital, pars orbitalis of the inferior frontal gyrus, precuneus, lobules IV and V of cerebellar hemisphere and insula.

**Figure 4:**
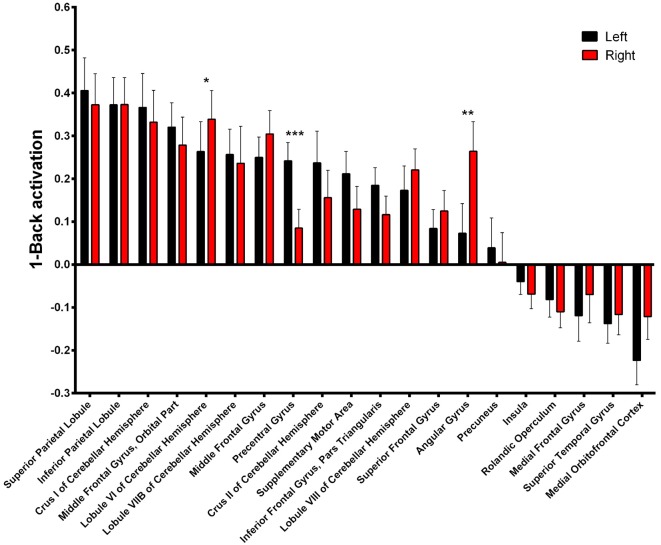
1-Back-related BOLD asymmetries. Comparison between left and right activations/deactivations during 1-Back performance. *α < 0.05, **α < 0.01, ***α < 0.001 in Bonferroni-corrected paired comparisons.

Asymmetry of the activated areas was analyzed by comparing left and right BOLD responses. During 1-Back performance (Figure 4, Supplementary Table S1), R > L activation was found in the lobule VI of the cerebellum (α = 0.016; *Z* = 3.331; Cohen’s *d* = −0.197) and in the angular gyrus (*α* = 0.010; *Z* = 3.469; Cohen’s *d* = −0.497). L > R activation occurred in the precentral gyrus (*α* < 0.001; *Z* = 4.527; Cohen’s *d* = 0.655).

**Figure 5:**
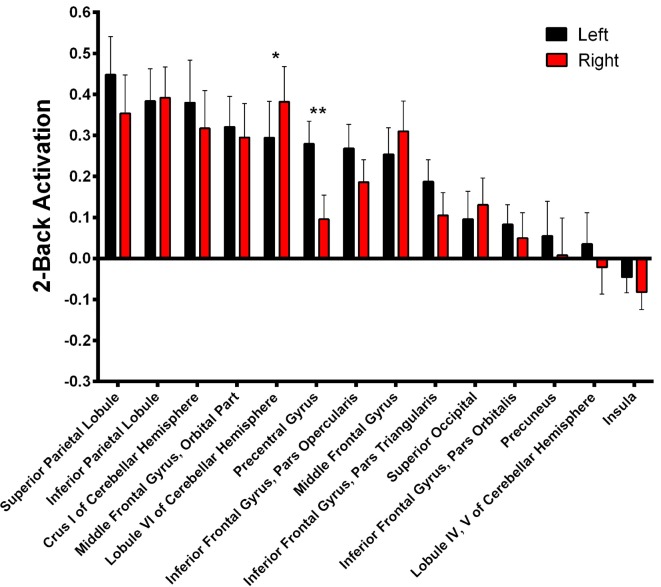
2-Back-related BOLD asymmetries. Comparison between left and right activations/deactivations during 2-Back performance. *α < 0.05, **α < 0.01 in Bonferroni-corrected paired comparisons.

During 2-Back trials (Figure 5, Supplementary Table S2) R > L activation could be identified in the lobule VI of the cerebellum *α* = 0.028; *Z* = 3.068; Cohen’s *d* = −0.215) while L > R activation was found in the precentral gyrus (*α* = 0.001; *Z* = 3.945; Cohen’s *d* = 0.685).

### Lateralized Activation in the Superior Parietal Lobule Is Associated With N-Back Performance

**Figure 6:**
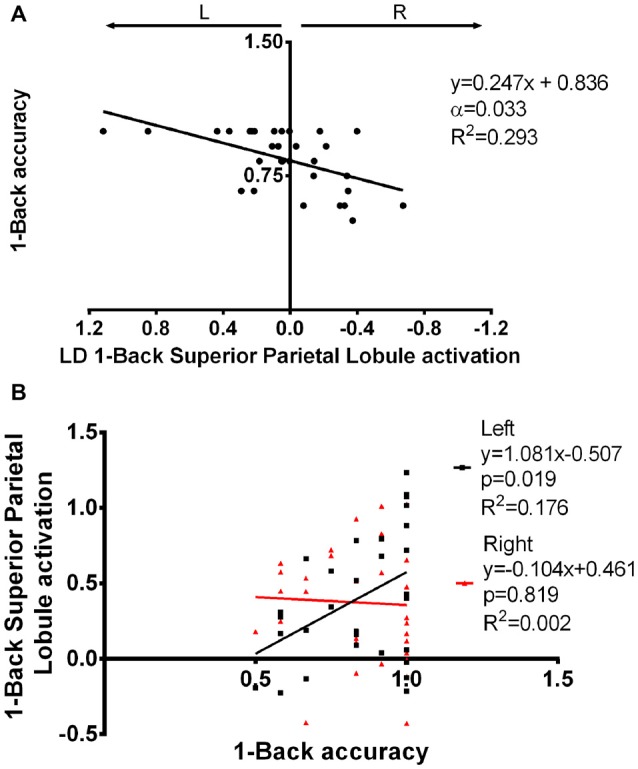
Superior parietal lobule’s LD in association with 1-Back accuracy.** (A)** Superior parietal lobule’s LD was positively correlated with 1-Back accuracy, i.e., increased left to right difference was associated with better performance. **(B)** The association seen in **(A)** seems to be mainly due to a positive correlation between left superior parietal lobule activation and task performance. LD, laterality degree; α, Bonferroni corrected *p*-value.

The impact of functional laterality on working memory was determined by establishing models with N-Back Acc as the dependent variable and LD as the independent variable. Superior parietal lobule 1-Back LD showed a positive correlation with Acc during this task. More specifically, we found that a leftward asymmetry in the activation of the superior parietal lobe was associated with better performance (α = 0.033; β = 0.247; *R*^2^ = 0.293—Figure 6A, Supplementary Table S3). Importantly, this association was maintained when age and education were added as co-variables in the regression model (*p* = 0.001; *β* = 0.270; *R*^2^ = 0.416). Simple 1-Back Acc vs. left or right BOLD response correlations showed that this association is mainly due to the left hemisphere contribution (Figure 6B).

No associations between performance and asymmetric activation of brain hemispheres were found in the 2-Back task.

## Discussion

In the present study we tested the hypothesis that task related BOLD response occurred in a lateralized fashion which could be associated with working memory performance in an older population. We demonstrated that the majority of the activated areas are similarly activated on the left and right hemispheres, although some asymmetries could be observed. Importantly, superior parietal lobule laterality was positively correlated with 1-Back performance.

The classic example of functional laterality is the well-known left lateralization for language (Toga and Thompson, 2003; Hugdahl, 2011), but degree of asymmetry has also been shown to predict performance in cognitive domains such as verbal and visuospatial ability (Gotts et al., 2013). Herein we evaluated functional lateralization during a verbal N-Back task (1- and 2-Back). The N-Back is a common tool for the study of working memory with a well-established pattern of cortical activation, which utilizes 0-Back as a reference task (Cohen et al., 1994; Owen et al., 2005; Yüksel et al., 2018). Our observations were in accordance with the expected pattern and recruited areas included the inferior parietal lobule, lateral cerebellum and prefrontal cortex (Owen et al., 2005). Asymmetrical activity however, was restricted to the lobule VI of the cerebellar hemisphere (R > L, 1- and 2-Back), precentral (L > R, 1- and 2-Back) and angular gyri (R > L, 1-Back).

The Hemispheric Asymmetry Reduction in Older Adults (HAROLD) model (Cabeza, 2002) postulates that high-performing older adults present compensatory bilateral activity. Cabeza et al. (2002) have shown that prefrontal cortex PET activation was more symmetrical in aged good performers when compared with young individuals or aged poor performers during a working memory task, suggesting a positive correlation between symmetry and performance. Even though these findings have been widely corroborated (Cabeza et al., 2004; Bergerbest et al., 2009; Piefke et al., 2012), some studies are still finding conflicting evidence (Vermeij et al., 2014; Brambilla et al., 2015) and more recently, the Compensation-Related Utilization of Neural Circuits Hypothesis (CRUNCH) was suggested (Reuter-Lorenz and Cappell, 2008; Schneider-Garces et al., 2010). This model postulates that this age-related symmetry is not necessarily due to homotopic activation, but results from an overall compensatory overactivation that happens not only in older adults, but also in younger subjects performing higher load cognitive tasks. In fact, from the 60 individuals initially included in our assay, only about 50% were able to perform the 2-Back task at an acceptable accuracy, indicating a high difficulty level for this specific population and therefore explaining the low number of lateralized areas.

Nonetheless, regarding the lateralized areas, both the right lateralization of the lobule VI of the cerebellar hemisphere and the precentral gyrus’s leftward bias may be associated with motor responses during the task. The latter is a classic motor area whose direction of activation follows the same trend found in the supplementary motor area in the 1-> 0-Back contrast, strongly suggesting an involvement of motor planning and/or execution. Additionally, left and right activation of the precentral gyrus seem similar in both presented contrasts, which is in accordance with previous reports of a load independent activation of the left sensorimotor cortex during the N-Back task (Jansma et al., 2000). Similarly, right but not left lobule VI of the cerebellar hemisphere is activated during right finger tapping (Stoodley and Schmahmann, 2009; Stoodley et al., 2010), although an involvement in working memory cannot be discarded as it was also previously reported as independent of motor performance both in an individual study (Stoodley et al., 2010) and in a meta-analysis (Stoodley and Schmahmann, 2009). Regarding the right > left asymmetry of the angular gyrus, this is the first report of functional lateralization in a working memory task. In fact, its activation in this function has been mostly regarded as bilateral (Carlson et al., 1998; Blokland et al., 2008). Nevertheless, this area has consistently shown lateralized activity in multiple other functions such as semantic and number processing, attention or spatial cognition (Seghier, 2013).

Interestingly, leftward asymmetry of the superior parietal lobule was correlated with better performance in the 1-Back task, which was mainly due to an association between left activation and accuracy. This area is known to be involved in working memory (Rottschy et al., 2013) and Koenigs et al. (2009) have previously found a lateralized effect, in which right lesions decreased working memory performance involving visuospatial manipulation. The authors were not able to find asymmetry effects in other types of working memory (including N-Back), which could be due to the low number of subjects (9 right vs. 4 left lesions). On the other hand, Otsuka et al. (2008) have determined that superior parietal lobule left activation was positively correlated with executive function while right activation was associated with short-term storage, further confirming the importance of laterality in this region.

In conclusion, herein we generated data confirming the areas activated during the N-Back task (Owen et al., 2005) and the reduced number of functionally lateralized areas, potentially due to age (Cabeza, 2002) or to high cognitive load (Reuter-Lorenz and Cappell, 2008). Importantly, lateralized areas were consistent in the two loads of working memory assessed and we were able to show for the first time a direct correlation between functional lateralization of an area and working memory performance. In fact, superior parietal lobule leftward lateralization was associated with improved accuracy in the 1-Back, highlighting the importance of left/right balance for ideal performance.

## Author Contributions

NCS and NS designed the cohort and coordinated the evaluations. ME and HL-A designed the study and wrote the first draft of the manuscript. CP-N recruited the subjects. TCC performed the neuropsychological tests. PM and JMS performed the fMRI acquisitions. ME, RM, PM, AA, NCS, NS and HL-A analyzed and interpreted the data.

## Conflict of Interest Statement

The authors declare that the research was conducted in the absence of any commercial or financial relationships that could be construed as a potential conflict of interest.

## Footnotes

^1^http://www.fil.ion.ucl.ac.uk/spm/software/spm8/
