# Supplementary material for: Functional Hemispheric (A)symmetries in the Aged Brain—Relevance for Working Memory
Source: Front Aging Neurosci. 2018 Mar 12;10:58. doi: 10.3389/fnagi.2018.00058 (PMC5857603; doi:10.3389/fnagi.2018.00058)
Supplement: Supplementary file 1 [file Table_1.DOCX]

|  | Left | | Right | | α | Z | Cohen's d |
| --- | --- | --- | --- | --- | --- | --- | --- |
|  | mean | SD | mean | SD |  |  |  |
| Superior Parietal Lobule | 0.406 | 0.425 | 0.373 | 0.401 | 2.173 | 0.255 | 0.080 |
| Inferior Parietal Lobule | 0.372 | 0.352 | 0.373 | 0.351 | 1.598 | 0.000 | -0.001 |
| Crus I of Cerebellar Hemisphere | 0.366 | 0.441 | 0.332 | 0.412 | 3.031 | 0.666 | 0.081 |
| Middle Frontal Gyrus, Orbital Part | 0.320 | 0.315 | 0.278 | 0.364 | 2.497 | 0.353 | 0.123 |
| Lobule VI of Cerebellar Hemisphere | 0.264 | 0.389 | 0.339 | 0.373 | 0.016 | 3.331 | -0.197 |
| Lobule VIIB of Cerebellar Hemisphere | 0.256 | 0.323 | 0.236 | 0.481 | 3.031 | 0.627 | 0.049 |
| Middle Frontal Gyrus | 0.250 | 0.265 | 0.304 | 0.304 | 1.517 | 1.529 | -0.192 |
| Precentral Gyrus | 0.242 | 0.236 | 0.085 | 0.243 | <0.001 | 4.527 | 0.655 |
| Crus II of Cerebellar Hemisphere | 0.237 | 0.412 | 0.156 | 0.356 | 0.623 | 2.038 | 0.210 |
| Supplementary Motor Area | 0.211 | 0.290 | 0.129 | 0.297 | 0.299 | 2.352 | 0.281 |
| Inferior Frontal Gyrus, Pars Triangularis | 0.185 | 0.229 | 0.116 | 0.241 | 0.218 | 2.489 | 0.291 |
| Lobule VIII of Cerebellar Hemisphere | 0.173 | 0.319 | 0.221 | 0.273 | 0.623 | 2.018 | -0.161 |
| Superior Frontal Gyrus | 0.084 | 0.247 | 0.125 | 0.266 | 1.827 | 1.333 | -0.160 |
| Angular Gyrus | 0.073 | 0.385 | 0.264 | 0.385 | 0.010 | 3.469 | -0.497 |
| Precuneus | 0.039 | 0.387 | 0.005 | 0.386 | 2.046 | 1.137 | 0.088 |
| Insula | -0.040 | 0.165 | -0.069 | 0.191 | 2.019 | 1.215 | 0.163 |
| Rolandic Operculum | -0.082 | 0.229 | -0.110 | 0.208 | 2.873 | 0.823 | 0.130 |
| Medial Frontal Gyrus | -0.119 | 0.330 | -0.070 | 0.366 | 1.558 | 1.470 | -0.141 |
| Superior Temporal Gyrus | -0.138 | 0.256 | -0.117 | 0.263 | 2.652 | 0.490 | -0.081 |
| Medial Orbitofrontal Cortex | -0.224 | 0.309 | -0.121 | 0.298 | 0.613 | 1.985 | -0.338 |

**Supplementary Table 1 - 1-Back Laterality statistics.**

Statistics for left vs right 1-Back BOLD response. SD=standard deviation; α=Bonferroni corrected p-value.
