# Supplementary material for: Functional Hemispheric (A)symmetries in the Aged Brain—Relevance for Working Memory
Source: Front Aging Neurosci. 2018 Mar 12;10:58. doi: 10.3389/fnagi.2018.00058 (PMC5857603; doi:10.3389/fnagi.2018.00058)
Supplement: Supplementary file 2 [file Table_2.DOCX]

|  | Left | | Right | | α | Z | Cohen's d |
| --- | --- | --- | --- | --- | --- | --- | --- |
|  | mean | SD | mean | SD |  |  |  |
| Superior Parietal Lobule | 0.448 | 0.434 | 0.354 | 0.437 | 1.457 | 1.023 | 0.216 |
| Inferior Parietal Lobule | 0.384 | 0.369 | 0.392 | 0.352 | 1.370 | 0.406 | -0.022 |
| Crus I of Cerebellar Hemisphere | 0.380 | 0.486 | 0.317 | 0.431 | 1.495 | 1.055 | 0.136 |
| Middle Frontal Gyrus, Orbital Part | 0.321 | 0.348 | 0.295 | 0.388 | 1.370 | 0.373 | 0.070 |
| Lobule VI of Cerebellar Hemisphere | 0.294 | 0.417 | 0.382 | 0.399 | 0.028 | 3.068 | -0.215 |
| Precentral Gyrus | 0.280 | 0.260 | 0.096 | 0.277 | 0.001 | 3.945 | 0.685 |
| Inferior Frontal Gyrus, Pars Opercularis | 0.269 | 0.272 | 0.186 | 0.258 | 0.265 | 2.289 | 0.312 |
| Middle Frontal Gyrus | 0.254 | 0.305 | 0.310 | 0.347 | 1.423 | 1.347 | -0.172 |
| Inferior Frontal Gyrus, Pars Triangularis | 0.188 | 0.250 | 0.105 | 0.260 | 0.265 | 2.256 | 0.322 |
| Superior Occipital | 0.096 | 0.315 | 0.131 | 0.307 | 1.226 | 0.893 | -0.110 |
| Inferior Frontal Gyrus, Pars Orbitalis | 0.083 | 0.227 | 0.050 | 0.290 | 1.744 | 1.153 | 0.127 |
| Precuneus | 0.055 | 0.394 | 0.008 | 0.423 | 1.180 | 1.510 | 0.115 |
| Lobule IV, V of Cerebellar Hemisphere | 0.036 | 0.357 | -0.021 | 0.307 | 0.883 | 1.704 | 0.171 |
| Insula | -0.046 | 0.177 | -0.082 | 0.202 | 1.744 | 1.153 | 0.191 |

**Supplementary Table 2 - 2-Back laterality statistics.** Statistics for left vs right 2-Back BOLD response. SD=standard deviation; α=Bonferroni corrected p-value.
