# Supplementary material for: Functional Hemispheric (A)symmetries in the Aged Brain—Relevance for Working Memory
Source: Front Aging Neurosci. 2018 Mar 12;10:58. doi: 10.3389/fnagi.2018.00058 (PMC5857603; doi:10.3389/fnagi.2018.00058)
Supplement: Supplementary file 3 [file Table_3.DOCX]

|  | p | α | β | R^2^ |
| --- | --- | --- | --- | --- |
| Superior Parietal Lobule | 0.002 | 0.033 | 0.247 | 0.293 |
| Inferior Parietal Lobule | 0.015 | 0.269 | 0.363 | 0.188 |
| Crus I of Cerebellar Hemisphere | 0.044 | 0.612 | -0.242 | 0.133 |
| Middle Frontal Gyrus, Orbital Part | 0.030 | 0.518 | 0.272 | 0.157 |
| Lobule VI of Cerebellar Hemisphere | 0.039 | 0.625 | -0.629 | 0.139 |
| Lobule VIIB of Cerebellar Hemisphere | 0.040 | 0.625 | -0.280 | 0.142 |
| Middle Frontal Gyrus | 0.161 | 1.770 | 0.266 | 0.067 |
| Precentral Gyrus | 0.009 | 0.163 | 0.536 | 0.215 |
| Crus II of Cerebellar Hemisphere | 0.257 | 2.316 | -0.161 | 0.044 |
| Supplementary Motor Area | 0.708 | 4.423 | 0.063 | 0.005 |
| Inferior Frontal Gyrus, Pars Triangularis | 0.835 | 2.395 | -0.041 | 0.002 |
| Lobule VIII of Cerebellar Hemisphere | 0.202 | 2.017 | -0.270 | 0.056 |
| Superior Frontal Gyrus | 0.110 | 1.434 | 0.363 | 0.086 |
| Angular Gyrus | 0.632 | 4.423 | -0.061 | 0.008 |
| Precuneus | 0.116 | 1.434 | 0.299 | 0.083 |
| Insula | 0.417 | 3.337 | 0.234 | 0.023 |
| Rolandic Operculum | 0.798 | 2.987 | 0.049 | 0.002 |
| Medial Frontal Gyrus | 0.725 | 4.248 | 0.068 | 0.004 |
| Superior Temporal Gyrus | 0.747 | 3.623 | -0.073 | 0.004 |
| Medial Orbitofrontal Cortex | 0.876 | 1.670 | -0.018 | 0.001 |

**Supplementary Table 3 - 1-Back accuracy correlations with BOLD laterality.** Statistics for 1-Back accuracy correlations with laterality degree. p=p-value ; α=Bonferroni corrected p-value.
